# MRI-based image-guided adaptive brachytherapy for locally advanced cervical cancer in clinical routine: a single-institution experience

**DOI:** 10.3389/pore.2025.1612077

**Published:** 2025-05-01

**Authors:** Zoltán Végváry, Renáta Kószó, Zsófia Együd, Linda Varga, Viktor Róbert Paczona, Adrienn Cserháti, Viorica Gal, Zoltán Varga, Zoltán Nagy, Bence Deák, Ferenc Borzák, Julianna Bontovics, Emese Fodor, Judit Oláh, Zsuzsanna Kahán

**Affiliations:** Department of Oncotherapy, University of Szeged, Szeged, Hungary

**Keywords:** cervical cancer, adaptive radiotherapy, image-guided brachytherapy, interstitial brachytherapy, MRI-based brachytherapy

## Abstract

**Background:**

MRI-based image-guided adaptive brachytherapy (IGABT) is a new approach for individual dose escalation and control of organ at risk (OAR) doses and toxicities in the treatment of locally advanced cervical cancer.

**Methods:**

Various radiotherapy-related parameters and the feasibility of the treatment based on acute toxicity were analyzed in a total of 50 cases in two cohorts who received a brachytherapy (BT) boost after definitive chemoradiotherapy with either an MRI-based IGABT technique (24 patients) or CT-only image guidance (26 patients). For target volume, OAR delineation, and dose prescription, the EMBRACE II protocol was followed.

**Results:**

The features of the target volumes and dose coverage did not differ between the two groups regarding teletherapy. At BT, however, while the High-Risk Clinical Target Volumes (CTVHR) did not differ the D90 dose coverage was significantly higher in the MRI-based IGABT group than in the non-MRI-based group (7.37 ± 0.55 Gy vs. 6.87 ± 0.84 Gy, p = 0.015). The CTVHR D98 doses showed a strong trend in favor of the MRI-based technique (6.16 ± 0.59 Gy, vs. 5.72 ± 0.95 Gy, p = 0.051). Cumulative doses to the CTVHR by means of both D90 and D98 were significantly higher in the MRI-based treatment group than the other group (86.64 ± 4.76 Gy vs. 81.56 ± 8.29 Gy, p = 0.011 and 77.23 ± 4.39 Gy vs. 73.40 ± 7.80 Gy, p = 0.037, respectively). Regarding OAR exposure, doses to the bladder, rectum, and sigmoid did not differ between the two cohorts.

**Conclusion:**

Our first clinical results support the implementation of IGABT as a key component of image-guided adaptive radiotherapy (IGART) aiming at tumor dose-escalation and OAR protection.

## Introduction

Cervical cancer continues to be a significant health issue and cause of cancer morbidity and mortality, with around 660,000 new cases and 350,000 deaths related to the disease globally each year and approximately 1,000 new cases and about 400 deaths in Hungary [1]. Of the many modern approaches, radiotherapy is one that may improve outcome. It is estimated that by implementing an image-guided adaptive radiotherapy (IGART) technique, 5-year disease-free survival will be more than 90% among patients with locoregional disease [2].

While early-stage cervical cancer can be cured with surgery or even with radiotherapy alone in certain clinical circumstances, the standard curative treatment for locally advanced cervical cancer is concomitant chemoradiotherapy (CRT) [3–8]. Intracavitary (IC) or combined intracavitary/interstitial (IC/IS) brachytherapy (BT) has been an essential component of the IGART approach in the last decade. No relevant clinical data indicate that BT boost could be replaced by any advanced external beam radiotherapy (EBRT) technique providing an equivalent outcome [9]. Due to excellent soft tissue resolution providing additional information regarding residual tumor extent, environmental propagation, and surrounding normal anatomy, magnetic resonance imaging (MRI)-based image-guided adaptive BT (IGABT) has become the gold standard curative treatment for locally advanced cervical cancer [2]. Since access to MRI has been limited in many clinical centers and its shortage is often aggravated by logistical and financial obstacles, computed tomography (CT)-based 3-dimensional (3D) image guidance is often used to replace MRI for BT planning.

Based on The Groupe Européen de Curiethérapie (GEC) and the European SocieTy for Radiotherapy & Oncology (ESTRO) recommendations, the widespread use of MRI-based image guidance has been receiving increasing clinical support in terms of both precision treatment planning and clinical outcome. The EMBRACE-I trial is considered to be the first prospective multicenter study offering a benchmark for the utilization of MRI-guided IGABT, with an excellent 92% overall 5-year local control rate [10]. Data from retroEMBRACE, a large retrospective multicenter analysis ongoing in parallel with EMBRACE-I, showed an 89% overall local control rate at 5 years [11]. Consequently, the EMBRACE-II was launched in 2016 as the first prospective multicenter interventional study aiming at uniform target concept and dose prescription protocol for MRI-guided IGABT to further improve efficiency and reduce treatment-related moderate and severe morbidity. A strict complex protocol of imaging, EBRT, and BT treatment planning and treatment delivery has been devoted to these aims. Since MRI-guided IGABT provides individually inhomogeneous dose distribution and the possibility of dose escalation in large and irregular volumes based on complex applicator arrangements and 3D treatment planning, this sophisticated technique ensures a superior outcome reinforced by the excellent clinical results from the previous EMBRACE studies [12].

We introduced the IGART technique in routine practice in our department at the end of 2015 for the definitive CRT of locally advanced cervical cancer. As a next step, we set out to introduce and adapt the recommendations of the EMBRACE-II protocol [2]. For various reasons, not all patients received MRI-based IGABT during the procedure. We intended to analyze our first findings on both the dosimetry and treatment-related toxicity aspects depending on the BT technique applied (MRI-based vs non-MRI based).

## Materials and methods

### Study characteristics

The research on IGART had been approved by the Human Investigation Review Board, University of Szeged, Albert Szent-Györgyi Clinical Centre (68/2015-SZTE), and all patients gave their informed consent to participate.

Radiotherapy-related parameters and the treatment’s feasibility based on acute toxicity were analyzed in two cohorts of patients: Group 1 patients received their BT boost after CRT with MRI-based IGABT technique between February 2017-January 2020 (MRI-based BT) and Group 2 had a BT boost supported with CT imaging only between February 2016-August 2022 (non-MRI-based BT) as part of the definitive treatment for locally advanced cervical cancer.

Inclusion criteria used the stage IB-IVA International Federation of Gynecology and Obstetrics (FIGO) 2009 system and included histologically proven squamous cell carcinoma, adeno-squamous carcinoma, or adenocarcinoma of the uterine cervix and the completion of curative CRT and BT. The main exclusion criteria were the presence of distant metastases, including metastatic para-aortic lymph nodes beyond the level of the lumbal 1-2 (L1-L2) vertebral interspace, or any non-compliance to the protocol.

All patients underwent a gynecologic examination, additional cystoscopy and/or rectoscopy if organ involvement was suspected, and abdomino-pelvic MRI for pelvic staging at baseline. A diagnostic positron emission tomography/CT (PET/CT) scan was performed for the accurate assessment of lymph node involvement and exclusion of distant metastases. A second pelvic MRI was done on the fifth week of CRT in all cases to assess tumor response before the BT boost. During the MRI, contrast medium in the vagina was applied for better visualization.

### Teletherapy technique

For EBRT, all patients went through similar treatment planning and delivery procedures. Patients were asked to empty their bowel before the treatment planning CT and each treatment session thereafter. Likewise, as a special bladder protocol, patients were instructed to urinate first, then drink 500 mL water in 30 min. Patients were positioned on the abdominal/pelvic module of the All In One (AIO) Solution (Orfit, Wijnegem, Belgium) and fixed with the Pelvicast System (ORFIT, Wijnegem, Belgium). Three series of non-contrast CT scans were taken (GE Healthcare Discovery™ RT CT, GE Healthcare, Chicago, IL, United States) from the top of the kidneys to the distal edge of the inguinal regions with an empty (0 min), comfortably filled (30 min), and full bladder (45 min).

Diagnostic T2 weighted (T2_w_) MRI and PET/CT scans were fused to the treatment planning CT for better target definition. The delineation of the volumes of interest was based on the guidelines of the EMBRACE-II study. (2,12) Two experienced radiologists (A.C. and V.G.) participated in the contouring procedures throughout the entire radiotherapy course. Dose prescription to these target volumes and the dose constraints of the indicated OARs are presented in Table 1. Cumulative EBRT + IGABT 2Gy/fraction equivalent dose (EQD2) was calculated with the linear quadratic model using α/β = 10 for target volumes and α/β = 3 value for OARs [13]. A total dose of 45 Gy was delivered to the PTV in 1.8 Gy/fraction daily doses. A simultaneously integrated boost (SIB) dose was applied to pathological lymph nodes to a total dose of 55 Gy if the parailiac lymph node region (PIL) was affected and 57.5 Gy if the para-aortic lymph node region (PAO) was affected. For dose reporting, the usual dose-volume data were utilized as indicated.

**TABLE 1:** Institutional dose prescription and dose constraints for applied EBRT.

EBRT		
Dose and volume parameter		Dose constraints
GTV_T_init	Volume	
CTV_T_HRinit	Volume	
CTV_T_LRinit	Volume	
CTV-E	Volume	
ITV45 (Gy)	D99.9	D_99.9%_ > 42,75Gy
	D98	
	D50	
GTV-N	NA	
PTV45 (Gy)	D98	
	V95	D_95%_ > 42,75Gy
CTV-N (Gy)	D98	D_98%_ > 55Gy
	Dmax	D_max_ < 58,85Gy
PTV-N (Gy)	D98	D_98%_ > 49,5Gy
Bowel (cm^3^)	V15Gy	
	V30Gy	V_30Gy_ < 500 cm^3^
	V40Gy	V_40Gy_ < 250 cm^3^
	Dmax	D_max_ < 47.25Gy
Sigmoid (%)	V30Gy	
	V40Gy	
	Dmax	D_max_ < 47.25Gy
Bladder (%)	V30Gy	V_30Gy_ < 80%
	V40Gy	V_40Gy_ < 60%
	Dmax	D_max_ < 47.25Gy
Rectum (%)	V30Gy	V_30Gy_ < 95%
	V40Gy	V_40Gy_ < 75%
	Dmax	D_max_ < 47.25Gy
Femoral heads	Dmax	D_max_ < 50Gy
Body (cm^3^)*	V43Gy	V_43Gy_/V_PTV45Gy_ < 1.15
	V50Gy	

Radiotherapy was delivered with a comfortably filled bladder according to the bladder protocol. In all cases, a 5-field or 7-field inverse intensity-modulated RT (IMRT) planning technique was applied using the Eclipse v13.6 planning system (Varian Oncology Systems, Palo Alto, CA, United States). Treatment was delivered with a Varian TrueBeamSTx or VitalBeam (Varian Oncology Systems, Palo Alto, CA, United States) linear accelerator equipped with high-definition (HD) 120 or Millenium120 multileaf collimator performing daily cone-beam CT (CBCT) verification and couch repositioning referring to bony anatomy.

### Concomitant chemotherapy

A total of five cycles of concomitant 40 mg/m^2^ cisplatin per week chemotherapy was delivered during the EBRT. Possible dose reduction or withholding was based on continuous monitoring of blood cells and kidney function using the calculation of estimated glomerular filtration rate (eGFR) (Cockcroft-Gault formula).

### BT technique

Following CRT, patients had three or four BT boost treatments, based on the cumulative target doses in the various target volumes and OAR dose constraints. If the risk of overlapping the hard dose constraint of the OAR at the fourth brachytherapy session was foreseen, the brachytherapy boost dose was delivered in three sessions only. The delivery of two fractions on consecutive days and another two fractions a week later was aimed at in order to complete the radiotherapy procedure within 50 days.

Before the BT session patients received an enema. A Foley catheter was inserted into the bladder and the bladder was filled with 50–100 mL of physiological saline. The volume was maintained during treatment to ensure reproducibility. In case of IC treatment, minor opiate tramadolor and muscle relaxant premedication were applied 20 min before application. For the IC/IS procedure, patients were provided epidural analgesia during the intervention, which was maintained if the applicators and the needles remained in the patient for the consecutive BT session the next day.

IC/IS BT was applied only in the MRI-based IGABT group if significant residual tumor volume or parametrial infiltration was present at the time of the BT. In the non-MRI-based BT cohort, patients were treated with the simple IC technique. CT- and MRI-compatible Ring or 3D Interstitial Ring Applicator sets (Varian Oncology Systems, Palo Alto, CA, United States) were used for all patients.

The maintenance of the geometry of the applicator towards the target volume was ensured by a tight vaginal packing aimed at pushing away the rectum and bladder and fixing the applicator against the cervix. The packing was filled with ultrasound gel to make it distinguishable from the vagina at planning. The applicator was also fixed with bandages to the patient. A special CT/MR-compatible stretcher (Spinal board BAR025, Fazzini, Italy) was used for patient transportation. The patient stayed on that device in a comfortable supine position throughout the procedure including verification and dose delivery.

After inserting the applicator(s) in all cases, a non-contrast CT series with 2.5 mm slice thickness was acquired for safety reasons and to check the applicator’s position. CT verification was used before brachytherapy delivery for safety, quality assurance, and planning purposes even if an MRI was not performed. Patients receiving MRI-based individualized IGABT were then transferred with the applicator *in situ* to the MRI unit before the first and third BT sessions at a minimum. In a few cases, T2_w_ fast spin echo (FSE) sequences were acquired with parallel orientation regarding the cervix uteri, with 1 mm slice thickness and without an interslice gap according to the Gyn GEC ESTRO MRI guidelines [14]. Thereafter, 3D T2_w_ sequences were established for MRI-based applicator reconstruction and delineation. CT and MRI images were fused with rigid registration of soft tissues for quality control and planning purposes. Adaptive target volume definition and treatment planning objectives were performed in accordance with the International Commission on Radiation Units and Measurements (ICRU) 89 recommendations [15]. The following BT target and OAR volumes were segmented: GTVres, CTVHR, bowel, bladder, and rectum. For dose recording, right and left Point A were indicated. Patients were prescribed cumulative doses; the dose constraints for the OARs are indicated in Table 2. CTVHR and GTV remained (GVTres) and OARs were delineated in an applicator *in situ* T2w MRI sequence. The latest treatment planning MRI was reused for contouring with superimposition if the succeeding second and/or fourth BT fraction was CT-based only.

**TABLE 2:** Cumulative doses aimed at during IGABT (*in MRI-based IGABT only).

Dose-volume parameter	Prescribed dose (EQD2 α/β = 10 Gy)	Dose constraint (EQD2, α/β = 3Gy)
D90 CTVHR	>85Gy	
D98 CTVHR	>75Gy	
D98 GTVres*	>90Gy	
Dose to Point A, left	to be recorded	
Dose to Point A, right	to be recorded	
DBowel 2 cm^3^		<75Gy
DBladder 2 cm^3^		<85Gy
DRectum 2 cm^3^		<75Gy
DSigmoid 2 cm^3^		<75Gy

In the non-MRI based BT group, non-contrast CT scans were used for delineation and previous (pre-radiotherapy and pre-BT) MRI images assisted contouring. CTVHR and OARs were delineated with adaptation to the GYN GEC-ESTRO guidelines, however, no residual GTV was contoured. MRI images acquired during the fifth week of EBRT helped target definition without performing CT-MRI fusion.

The BrachyVision TPS v13.6 planning system (Varian Oncology Systems, Palo Alto, CA, United States) was utilized for treatment planning. During optimization, first the built-in automatic option of the planning system was used, and then, taking into account the unique anatomical conditions, the plan was manually refined in accordance with the dosimetric goals. All patients were treated with a Varian Gammamed Plus iX (Varian Oncology Systems, Palo Alto, CA, United States) high dose-rate (HDR) afterloader.

### Toxicity registration

Treatment-related acute morbidity was registered on a weekly basis according to the Common Terminology Criteria for Adverse Events (CTCAE v. 4.0). Clinical data on treatment-related acute skin, gastro-intestinal, genito-urinary, or hematologic toxicity and kidney function impairment was collected, and the worst toxicity grade within a category was counted in each case.

### MRI-based quantitative tumor regression evaluation

**FIGURE 1:**
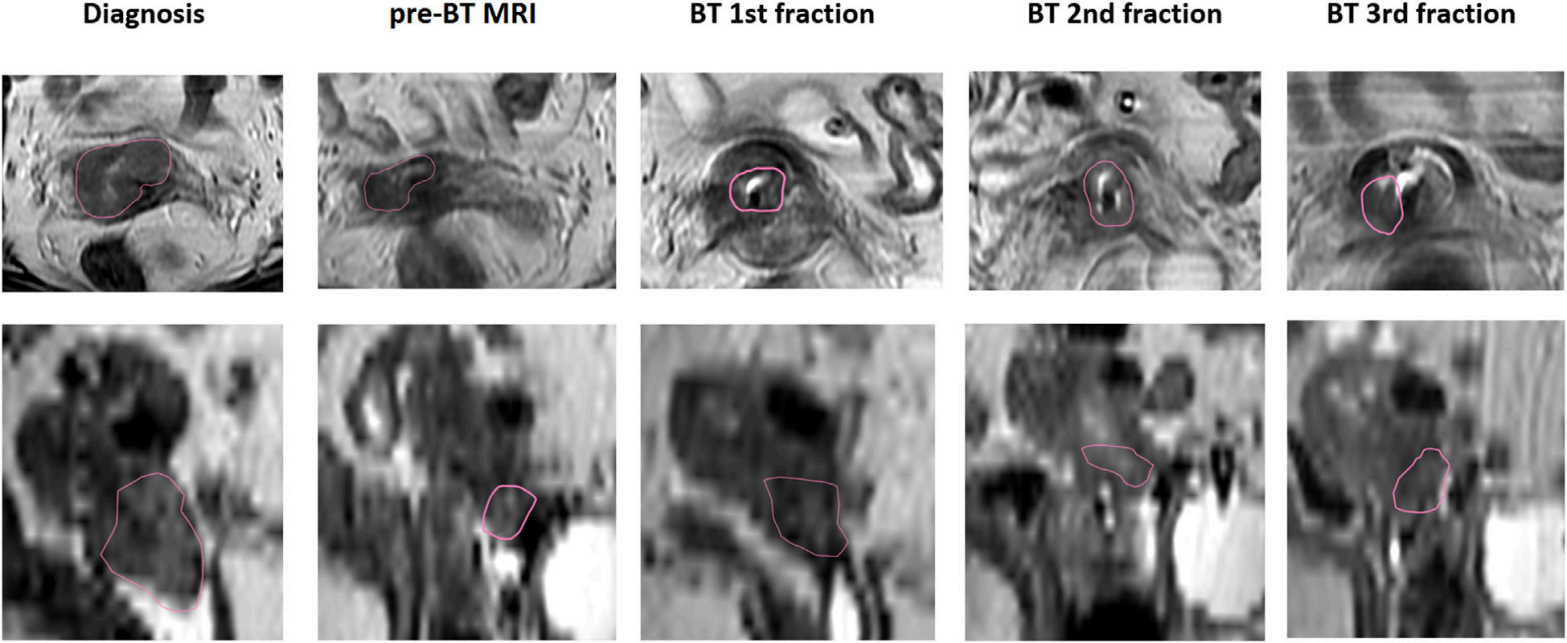
MRI-based GTV evaluation during treatment, transversal and sagittal views (red contour indicates the GTV).

**FIGURE 2:**
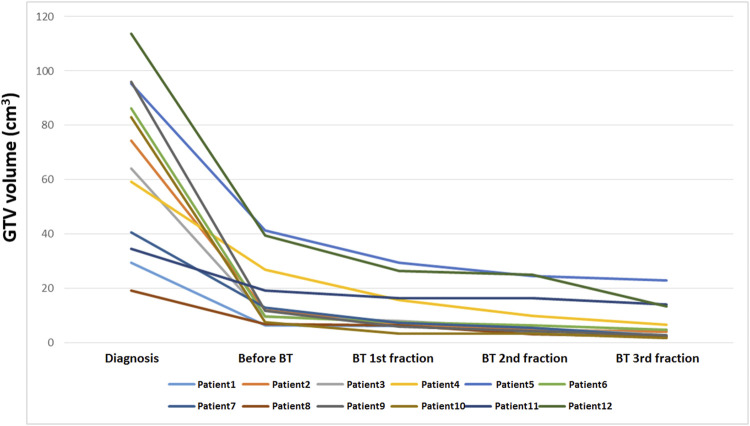
Quantitative MRI-based tumor regression analysis during treatment (n = 12).

Based on the T2w MR image series, a quantitative tumor regression analysis was performed for the MRI-based IGABT cohort during the treatment process. The GTV-Tinit was evaluated in the first diagnostic MRI while the GTVres volumes were assessed in both the pre-BT MRI and subsequent applicator *in situ* MRI sequences (Figures 1, 2).

### Statistical analysis

Continuous data were expressed as mean ± standard deviation (SD) values if appropriate. Tumor characteristics in the two cohorts were compared with an independent sample t-test for the continuous and Chi-squared test for the categorical variables. An independent sample t-test was used to analyze the various dose parameters between the treatment groups. Statistical software IBM SPSS statistics version 26.0 (SPSS Inc., Chicago, IL, United States) was used for statistical analysis. P-values <0.05 were regarded as statistically significant.

## Results

The clinical data of 50 locally advanced cervical cancer patients treated with definitive CRT and IGABT boost between February 2016 and August 2022 at the Department of Oncotherapy University of Szeged were evaluated in our retrospective study. The average (±SD) age of the patients overall was 52.7 ± 12.6 years, ranging between 26 and 83 years. The median (±SD) overall treatment time was 53.8 ± 9.9 (range: 42–81) days. All patients had FIGO IB-IIIB stage locally advanced cervical cancer, 22 (44.0%) of them had negative lymph node status, and 28 (56.0%) were diagnosed with N1 disease (Table 3). The vast majority of cases, 43 patients (86.0%), had squamous cell carcinoma, while seven patients (14.0%) had adenocarcinoma. Two-thirds of the study population was diagnosed with grade 2-3 tumors (Table 3). Of the 50 patients, 43 (86.0%) completed the scheduled five chemotherapy cycles, while seven (14.0%) received only four cycles of cisplatin. In the MRI-based IGABT group, among 24 patients, eight received combined the IC/IS IGABT treatment. Altogether, six patients had three brachytherapy sessions only due to dose limitations to the OARs: one in the MRI-based IGABT group and five in the CT-based treatment group. Histology, tumor grade, FIGO, and lymph node stage were well balanced between the two treatment groups. Relevant tumor characteristics at the time of EBRT are presented in Table 3.

**TABLE 3:** Characteristics of cases (stage, pathology, teletherapy, and chemotherapy).

		MRI-based BT	Non-MRI-based BT	p
Stage	cT1b cT2a cT2b cT3b	4 2 17 1	3 4 15 4	0.447
	cN0 cN1	10 14	12 14	0.783
	FIGO Ib IIa IIb IIIb	6 1 16 1	3 4 16 3	0.293
Pathology	Histology Squamos cell carcinoma Adenocarcinoma	22 2	21 5	0.420
	Grade I II III NA	2 11 6 5	3 10 5 8	0.856
Teletherapy features	GTVinit (cm3) (mean ± SD, range)	86.02 ± 49.68, 3.1–176.7	99.40 ± 43.50, 43.9–192.3	0.310
	GTVN (n = 13 vs. 13, cm3) (mean ± SD, range)	8.63 ± 6.81, 1.3–22.4	6.88 ± 5.64, 2.1–19.9	0.483
	PTV45Gy (cm3) (mean ± SD, range)	1,335.0 ± 187.1, 1,083.4–1791.3	1,435.9 ± 216.6, 1,123.1–1894.0	0.086
	PTVN (n = 12 vs. 13, cm3) (mean ± SD, range)	63.46 ± 63.43, 15.9–193.6	45.05 ± 25.21, 15.1–109.3	0.363
	Small pelvis (n, %)	0, 0%	0, 0%	0.751
	Large pelvis (n, %)	17, 70.8%	20, 76.9%	
	Large pelvis + PAN (n, %)	7, 29.2%	6, 23.1%	
Chemotherapy	Number of cycles (mean ± SD, range)	4.88 ± 0.32, 4–5	4.85 ± 0.37, 4–5	0.775

The features of the target volumes did not differ between the two groups; using the same EBRT technique, no significant difference was found between the coverage of PTV45, PTV-N, CTV-N_PIL, or CTV-N_PAO of the two groups, as shown in Table 4.

**TABLE 4:** Delivered doses to target volumes during teletherapy.

		MRI-based BT	Non-MRI-based BT	p
PTV45	V95% (%)	95.6 ± 2.3	95.7 ± 1.4	0.791
	Dmax (Gy)	55.4 ± 12.6	52.5 ± 5.6	0.275
PTVN	D98% (%)	91.0 ± 11.3	97.1 ± 3.0	0.187
	Dmax (Gy)	57.8 ± 1.6	56.7 ± 3.7	0.387
CTVN_PIL	D98% (Gy)	55.0 ± 2.3	55.0 ± 1.6	0.975
CTVN_PAO	D98% (Gy)	44.9 ± 2.8	43.8 ± 0.7	0.181

While the CTVHR volumes were similar, the D90 dose to them at BT was significantly higher in the MRI-based IGABT group than in the non-MRI-based group; the CTVHR D98 doses showed a strong trend in favor of the MRI-based technique (Tables 5, 6). Likewise, the cumulative doses to the CTVHR by means of both D90 and D98 were significantly higher in the MRI-based treatment group than in the other group (Table 7). The Point A target doses, however, did not differ between the two groups (Table 6). In the MRI-based IGABT group, an average EQD2 D98 dose of 8.01 ± 1.15 (5.3–10.2) Gy per BT session and 90.08 ± 11.99 Gy cumulative dose was delivered to the GTVres (Tables 6, 7). When the doses to the CTVHR and GTVres within the MRI-based IGABT group were compared according to the BT technique, no difference was found between the IC only and IC/IS cases.

**TABLE 5:** Target volumes during brachytherapy.

	MRI-based BT (mean ± SD, range)	Non-MRI-based BT (mean ± SD, range)	p
CTVHR (cm^3^)	28.91 ± 11.49, 14.3–64.8	34.36 ± 12.97, 14.8–73.5	0.123
GTVres (cm^3^)	6.76 ± 5.41, 1.1–22.2	NA	NA

**TABLE 6:** Delivered doses to target volumes or reference points during the brachytherapy sessions.

	MRI-based BT (mean ± SD, range)	Non-MRI-based BT (mean ± SD, range)	p
D90 CTVHR (Gy)	7.37 ± 0.55, 5.7–8.3	6.87 ± 0.84, 5.0–7.8	0.015
D98 CTVHR (Gy)	6.16 ± 0.59, 4.8–7.0	5.72 ± 0.95, 3.6–6.8	0.051
D98 GTVres (Gy)	8.01 ± 1.15, 5.3–10.2	NA	NA
Point A left (Gy)	5.82 ± 1.19, 3.7–9.3	5.71 ± 0.91, 4.2–8.5	0.716
Point A right (Gy)	5.54 ± 1.14, 3.8–8.5	5.44 ± 0.74, 4.3–6.6	0.714

**TABLE 7:** Delivered cumulative doses to target volumes during teletherapy plus brachytherapy (EQD2, α/β = 10).

	MRI-based BT (mean ± SD, range)	Non-MRI-based BT (mean ± SD, range)	p
D90 CTVHR (Gy)	86.64 ± 4.76, 74.00–95.09	81.56 ± 8.29, 64.08–90.74	0.011
D98 CTVHR (Gy)	77.23 ± 4.39, 67.85–84.03	73.40 ± 7.80, 58.30–82.53	0.037
D98 GTVres (Gy)	90.08 ± 11.99, 71.59–113.12	NA	NA

**FIGURE 3:**
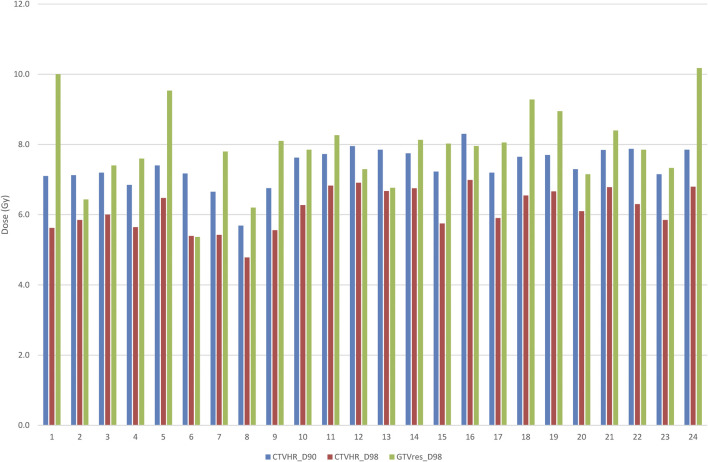
Individual target volume dose coverage in the MRI-based IGABT group (n = 24).

Next, the same dosimetry parameters of the MRI-based IGABT cohort were studied in chronological order; an apparent increase of doses by time was found (Figure 3). The prespecified OAR doses (the EQD2 doses to 0.1 cm^3^ and 2.0 cm^3^ volumes of the bladder, rectum, sigmoid, and bowel) are demonstrated in Tables 8, 9. The doses to the bladder, rectum, and sigmoid did not differ between the two groups. Interestingly, significantly less dose to the bowel was experienced in Group 2, both if the dose per BT fraction and the cumulative dose after EBRT and BT was considered (Tables 8, 9). OAR doses in the MR-based IGABT cohort did not fluctuate over time.

**TABLE 8:** Dose exposure to the various OARs at single brachytherapy sessions.

	D0.1 cm^3^ (mean ± SD, range)			D2cm^3^ (mean ± SD, range)		
	MRI-based BT	non-MRI based BT	p	MRI-based BT	non-MRI-based BT	p
Bladder (Gy)	7.49±.1.20, 4.5–9.4	8.10 ± 1.24, 6.4–12.8	0.084	5.52 ± 0.97, 2.8–6.4	5.74 ± 0.72, 4.5–7.6	0.373
Rectum (Gy)	5.61 ± 1.41, 2.4–8.2	6.04 ± 1.94, 2.0–9.5	0.379	3.86 ± 0.89, 1.7–5.0	4.03 ± 1.23, 1.5–5.9	0.597
Sigmoid (Gy)	5.61 ± 1.21, 3.7–8.9	5.48 ± 2.02, 0.0–9.5	0.785	3.83 ± 0.68, 2.7–5.0	3.75 ± 1.29, 0.0–5.9	0.793
Bowel (Gy)	5.34 ± 2.27, 0.0–8.3	3.65 ± 2.62, 0.0–7.4	0.019	3.73 ± 1.56, 0.0–5.3	2.52 ± 1.82, 0.0–5.0	0.015

**TABLE 9:** Delivered cumulative doses to OARs during the entire course of teletherapy plus brachytherapy (EQD2, α/β = 3).

	D0.1 cm^3^ (mean ± SD, range)			D2cm^3^ (mean ± SD, range)		
	MRI-based BT	non-MRI based BT	p	MRI-based BT	non-MRI-based BT	p
Bladder (Gy)	107.30±.17.19, 68.00–136.9	112.08 ± 16.08, 86.17–167.3	0.315	81.53 ± 10.07, 56.58–91.32	81.63 ± 7.22, 63.94–92.57	0.968
Rectum (Gy)	85.32 ± 16.88, 53.87–118.14	87.13 ± 18.78, 51.04–125.32	0.722	65.22 ± 7.57, 49.41–75.31	66.07 ± 8.79, 48.73–80.59	0.715
Sigmoid (Gy)	83.33 ± 14.36, 63.40–127.93	81.74 ± 19.55, 43.20–129.35	0.746	64.64 ± 5.66, 55.86–75.67	63.86 ± 9.06, 43.20–76.51	0.720
Bowel (Gy)	83.92 ± 20.08, 43.20–120.45	69.23 ± 21.19, 43.2–105.38	0.015	65.72 ± 10.49, 43.20–78.31	57.36 ± 11.51, 43.20–74.95	0.010

Regarding treatment-related side effects, the most frequent acute toxicity of any grade was nausea, leucopenia, and neutropenia in both groups. Interestingly, thrombocytopenia of grade 1 occurred significantly more in Group 2 (Table 10). There were no serious grade 3–4 adverse events registered (Table 10). No cessation of the curative treatment course was necessary due to severe toxicity.

**TABLE 10:** Treatment induced acute toxicity (CTCAE v. 4.0).

	MRI-based BT (n = 24) (n,%)			Non-MRI-based BT (n = 26) (n,%)			p
	Grade I	Grade II	Grade III	Grade I	Grade II	Grade III	
Leucopenia	4 (16.7%)	11 (45.8%)	4 (16.7%)	7 (26.9%)	11 (42.3%)	1 (3.8%)	0.411
Neutropenia	3 (12.5%)	9 (37.5%)	1 (4.2%)	6 (23.1%)	4 (15.4%)	1 (3.8%)	0.325
Anemia	9 (37.5%)	7 (29.2%)	0 (0%)	14 (53.8%)	5 (19.2%)	0 (0%)	0.494
Thrombocytopenia	6 (25.0%)	3 (12.5%)	0 (0%)	18 (69.2%)	0 (0%)	0 (0%)	0.040
Nausea	19 (79.2%)	0 (0%)	0 (0%)	13 (50.0%)	4 (15.4%)	1 (3.8%)	0.081
Vomitus	11 (45.8%)	1 (4.2%)	0 (0%)	9 (34.6%)	2 (7.7%)	0 (0%)	0.674
Enteritis	10 (41.7%)	0 (0%)	0 (0%)	13 (50.0%)	4 (15.4%)	0 (0%)	0.067
Proctitis	1 (4.2%)	0 (0%)	0 (0%)	2 (7.7%)	0 (0%)	0 (0%)	0.969
Cystitis	3 (12.4%)	1 (4.2%)	0 (0%)	1 (3.8%)	1 (3.8%)	0 (0%)	0.526
Incontinency	0 (0%)	0 (0%)	0 (0%)	0 (0%)	0 (0%)	0 (0%)	NA
Vaginitis	0 (0%)	0 (0%)	0 (0%)	2 (7.7%)	0 (0%)	0 (0%)	0.491
Dermatitis	0 (0%)	0 (0%)	0 (0%)	1 (3.8%)	0 (0%)	0 (0%)	0.978
Nephrotoxicity	3 (12.4%)	0 (0%)	0 (0%)	4 (15.4%)	0 (0%)	0 (0%)	0.875

## Discussion

In this exploratory analysis of our first clinical experience, we found that the MRI-based IGABT technique is appropriate both for individual dose escalation and control of OAR doses and toxicities in locally advanced cervical cancer. This should be the preferred approach, especially in cases with large and/or irregular extent post-teletherapy residual tumors.

The studied IGABT technique based on multimodality imaging, modern radiobiology knowledge, and novel teletherapy and BT technologies is a rational approach but requires effort from the whole medical staff to adopt special skills and make a series of labor-intensive key steps in patient-management. For this new strategy, invaluable help is provided by the published protocol of the EMBRACE-II international clinical study [16]. This book of precision on CRT and IGABT provides information on all challenges, including complex patient management, 3D image guidance, delineation and treatment planning, and proper BT applicator selection and arrangement. Furthermore, the EMBRACE team and Gyn GEC ESTRO collaborative group have been making efforts in the last two decades to disseminate the fundamentals of the aforementioned comprehensive concept through special training programs and within the frame of the ongoing EMBRACE-II international multi-center clinical study [17].

Due to the progression attributes of cervical carcinoma, sufficient loco-regional dose delivery is essential for tumor control. The review of Tanderup et al based on a large amount of clinical data demonstrated a strong correlation between the delivered dose to the target volumes and clinical outcome [18]. The importance of dose escalation is also supported by the results of the EBRACE-I study, achieving an excellent 5-year local control rate of 92% for the overall patient cohort [10]. Meanwhile, the complete remission rate was 98%, and only 98/1,318 patients (7.4%) had local failure in the entire study population. Notably, 90% of these cases showed relapse within the BT target volume only. According to the report of Schmid et al, higher local failure rates were associated with the presence of tumor necrosis, uterine corpus or mesorectal space infiltration, or a larger initial tumor extent (CTVHR > 45 cm^3^) [19]. Moreover, an adenocarcinoma or adenosquamos carcinoma histology subtype was associated with a significantly higher risk of local failure. Delivering 85 Gy D90 dose to the CTVHR resulted in a 95% local control for squamous cell carcinomas vs. only 86% for adenocarcinoma/adenosquamous carcinoma cases at 3 years. In accordance with the retroEMBRACE data, overall treatment time (OTT) was also found to be an independent risk factor on clinical outcome [19]. It is envisioned that the prospective follow-up data of EMBRACE II will not only point to the role of radiation dose in improved outcome but will demonstrate the feasibility of individually chosen dosing according to stage and tumor-related biomarkers in line with the importance of long-term quality of life (QOL) consideration [2]. In other words, dose escalation and dose de-escalation will become individually chosen strategies in modern practice.

Our first series of patients and clinical data in this paper represent the efforts and learning period we went through during the integration of the modern IGART technique into our institutional practice. First, we systematically introduced the bladder protocol and rigid image fusion of the multiple series of treatment planning CT and diagnostic pelvic MRI and PET/CT scans for EBRT contouring, including the recommended complex volume of interest template, into the planning system. Accordingly, all patients received risk-adapted elective pelvic irradiation and SIB to pathological lymph nodes if applicable using IMRT planning aims; daily cone-beam CT verification with correction to bony anatomy allowed for halving former PTV-CTV margins of 10 mm. Great attention was given to both teletherapy and chemotherapy as the basis of high-dose delivery at IGABT due to down-staging. Regarding the implementation of MRI-guided IGABT, the greatest challenges were ensuring the infrastructure of anesthesia, appropriate patient transport, adequate MRI imaging, and applicator reconstruction during the first couple of combined IC/IS treatment sessions. Handling the early target delineation and planning optimization uncertainties, we could gradually improve the BT target dose utilizing MRI guidance, resulting in a mean dose to the D90 CTVHR higher than 85 Gy. Although in the first few cases our main goal was to safely remain within the limits of OAR hard-dose constraints, later on we simultaneously aimed at the fulfillment of OAR soft-dose constraints and the planning aims of D90 CTVHR (90–95 Gy) and D98 GTVres (>95 Gy). The only explanation why the target dose was higher in the MRI-guided IGABT group could be that by having had identified the residual tumor and delivered a prespecified radiation dose to it, the dose to the CTVHR was unintentionally increased too. Our experience on eight cases (16,0%) having received BT with interstitial needles was favorable, indicating that the combined IC/IS technique is the adequate solution to serve this dual goal. The analysis in the retroEMBRACE study suggested that the simple IC BT technique has limitations in CTVHR dose coverage, especially in cases with larger residual tumor volumes (CTVHR > 30 cm^3^), hence resulting in suboptimal local control [20]. Another retroEMBRACE analysis by Fokdal et al. demonstrated that by using the combined IC/IS technique, the D90 CVTHR dose could be elevated by 9 Gy EQD_2_ and consequently significantly higher local control rates were found in patients with residual CTVHR > 30 cm^3^ volumes without increasing toxicity as compared to the use of the sole IC BT technique [21]. In fact, dedicated IC/IS applicators represent an excellent tool for the implementation of personalized treatment plans with unique complexity, allowing appropriate target coverage even if large residual tumors or parametrial infiltration are present [22, 23].

We have prospectively collected a set of acute toxicity measures possibly related to CRT; these were easily controlled and no serious side effects resulting in the cessation of therapy occurred. Due to the short follow-up time, the analysis of late toxicity is out of scope of this paper. Regarding long-term toxicity related to the described modern approach, the association between the delivered dose and the risk of moderate to severe chronic toxicity is highly supported by the results of the EMBRACEh-I study. In terms of urinary morbidity, the dose to the bladder of D2cm^3^ was correlated with the risk of grade 2 or higher fistula, cystitis, or bleeding; an increase of that dose from 75 Gy to 80 Gy almost doubled the risk of grade ≥2 cystitis [24]. Beyond rectal and bowel D2cm^3^ BT doses, the incidence of grade ≥2 diarrhea was associated with higher prescribed EBRT doses (45 vs. 50 Gy), total body V43 Gy values, and pathological lymph node SIB volumes [24]. Attention to vaginal toxicity has recently come into view [25]. Vaginal doses show extreme diversity depending on both the teletherapy and BT techniques. Vaginal dose de-escalation is crucial for the prevention of grade ≥2 vaginal stenosis; the EMBRACE II protocol puts great emphasis on the documentation and control of the vaginal dose, assuming a maximum dose of 65 Gy EBRT + BT EQD2 to the ICRU recto-vaginal point. In addition, the doses to the mid and lower parts of the vagina must be assessed using the Posterior-Inferior Border of Symphysis (PIBS) dose points [26]. It seems advantageous to use a ring and tandem applicator instead of a tandem and ovoids for this vaginal dose de-escalation approach [27] Interestingly, Mohamed et al. demonstrated that significant vaginal dose de-escalation may be achieved by reducing the dwell times in the ring or ovoids while increasing that in the tandem/interstitial needles without compromising the D90 CTVHR ≥85 Gy target dose [28].

Despite the excellent local control rates with the systematic utilization of IGART, with cases of advanced-stage disease, distant recurrence has still been a significant problem in light of the modest overall survival (OS) data. As a result, the need for progress has emerged recently in the field of novel systemic therapies. Currently, many clinical trials are ongoing using immunotherapy in addition to the standard CRT approach. Human papillomavirus (HPV)-associated cervical cancers are considered to be immunogenic tumors with increased neoantigen formation, high somatic mutation rate, and enhanced programmed death-ligand 1 (PD-L1) expression [29, 30]. Indeed, immunotherapy is now a part of standard care of metastatic/recurrent cervical carcinoma. Interestingly, the first phase III CALLA study failed to meet its primary endpoint when adding durvalumab to standard CRT in locoregional cervical carcinoma; progression-free survival (PFS) was not improved in the investigational arm [31]. In contrast, the phase III ENGOT-cx11/KEYNOTE-A18 study - beyond PFS advantage - resulted in a significant increase in OS: at 3 years OS was 82.6% for pembrolizumab plus CRT vs 74.8% for CRT alone [32]. Another approach, the use of induction chemotherapy prior to CRT, was tested in the phase III GCIG INTERLACE trial; a significant 9% PFS and 7% OS absolute benefit was demonstrated at 5 years of follow-up in favor of induction chemotherapy followed by CRT compared to standard CRT alone [33]. The results of two later studies are encouraging and will probably exert a great impact on the standard of care of locally advanced cervical cancer in the near future. In addition, beyond traditional risk evaluation, the integration of biomarkers serving tailored management is awaited; notably, molecular biomarkers are being investigated in a Translational Research sub-study by EMBRACE as well [34].

The protocol used by us has been widely used within and outside of the EMBRACE network. In accordance with our modest experience, a report on a larger experience of 392 patients over 10 years has been published [35].

In summary, we found the IGABT approach as a key element of IGART feasible and adequate for tumor dose-escalation and OAR protection at the same time in the management of cervical carcinoma.

## Data Availability

The raw data supporting the conclusions of this article will be made available by the authors, without undue reservation.
